# Dominance rank is associated with body condition in outdoor-living domestic horses (*Equus caballus*)

**DOI:** 10.1016/j.applanim.2015.02.019

**Published:** 2015-05

**Authors:** Sarah L. Giles, Christine J. Nicol, Patricia A. Harris, Sean A. Rands

**Affiliations:** a University of Bristol, School of Veterinary Science, Langford, Bristol BS40 5DU, UK; b University of Bristol, School of Biological Sciences, Tyndall Avenue, Bristol BS8 1TQ, UK; c WALTHAM Centre for Pet Nutrition, Equine Studies Group, Freeby Lane, Waltham-on-the-Wolds, Melton Mowbray, Leicestershire LE14 4RT, UK

**Keywords:** Equine, Fatness, Obesity, Social behaviour, Displacement

## Abstract

The aim of our study was to explore the association between dominance rank and body condition in outdoor group-living domestic horses, *Equus caballus*.

Social interactions were recorded using a video camera during a feeding test, applied to 203 horses in 42 herds. Dominance rank was assigned to 194 individuals. The outcome variable body condition score (BCS) was recorded using a 9-point scale. The variables age and height were recorded and considered as potential confounders or effect modifiers. Results were analysed using multivariable linear and logistic regression techniques, controlling for herd group as a random effect.

More dominant (*p* = 0.001) individuals generally had a higher body condition score (*p* = 0.001) and this association was entirely independent of age and height. In addition, a greater proportion of dominant individuals fell into the obese category (BCS ≥ 7/9, *p* = 0.005).

There were more displacement encounters and a greater level of interactivity in herds that had less variation in age and height, lending strength to the hypothesis that phenotypic variation may aid cohesion in group-living species. In addition there was a strong quadratic relationship between age and dominance rank (*p* < 0.001), where middle-aged individuals were most likely to be dominant. These results are the first to link behavioural predictors to body condition and obesity status in horses and should prompt the future consideration of behavioural and social factors when evaluating clinical disease risk in group-living animals.

## Highlights

• Body condition was associated with dominance rank.
• Food-dominant animals were more likely to be in the obese body condition category.
• Association of dominance and body condition was independent of age and height.
• Animals of an intermediate age were more likely to have a higher dominance rank.
• Herds more similar in height and age were more interactive.

## 1 Introduction

Broadly, dominance refers to an ordering between group-living animals, not necessarily linear (Appleby, 1983), which dictates the priority of access to resources. Drews (1993) defines this neatly as: “an attribute of the pattern of repeated, agnostic interactions, between two individuals, characterised by a consistent outcome and default response rather than escalation of conflict”. Dominance behaviours have evolved as a means of peaceful and quick conflict resolution in group-living animals (Bernstein, 1981; Drews, 1993; Hemelrijk, 2000). Group living is an important strategy for some grazing herbivorous species such as horses, who rely on vigilance and flight as a means of avoiding predation (Hamilton, 1971; Krause and Ruxton, 2002).

Individual animals within a group react and behave differently in any given situation, pursuing the best social strategy with relation to other herd members (Maynard-Smith and Parker, 1976; Krüger and Flauger, 2008). Thus different individuals may derive different costs and benefits from group membership (Alexander, 1974). These individual differences in social behaviours mean that in a stable, unchanging group, conflict is minimised during competition over a valued resource such as food. Conflict can have major fitness costs to the individuals involved and in addition could threaten the integrity of the group as a whole (Krause and Ruxton, 2002).

Depending on the resource, different social orders may be apparent in group living animals (Lee, 1983). In horses, dominance is known to be highly context specific. For instance, an individual that is dominant in a food-related context might not be given the same priority when determining access to a different resource such as shelter (Kiley-Worthington, 1990). Dominance in horses is also known to vary over time and between different seasons (Keiper and Sambraus, 1986). Dominance in our study refers purely to context-specific food-related dominance at an instantaneous moment in time, and will be defined as “an asymmetry in the outcome of dyadic interactions between individuals, or a priority of access to resources” (Drews, 1993).

Several studies on horses have noted an association between social status (in terms of dominance or aggression) and physical attributes. These physical attributes have included weight or size (Clutton-Brock et al., 1976; Berger, 1977; Houpt et al., 1978; Rutberg and Greenberg, 1990; Ingólfsdóttir and Sigurjónsdóttir, 2008), height (Rutberg and Greenberg, 1990), age (Keiper, 1988; Rutberg and Greenberg, 1990; Van Dierendonck et al., 1995; Sigurjonsdottir et al., 2003; Heitor et al., 2006; Ingólfsdóttir and Sigurjónsdóttir, 2008) and sex (Houpt and Keiper, 1982). Dominance has also been associated with the amount of time an individual has been resident in the herd (Clutton-Brock et al., 1976; Van Dierendonck et al., 1995).

There are a few examples of a positive association between dominance and body condition in other group living herbivorous ungulates such as fallow deer *Dama dama* (Jennings et al., 2010), bison *Bison bison* (Vervaecke et al., 2005) and cattle *Bos taurus* (Clutton-Brock et al., 1976; Šárová et al., 2013). However, no previous studies on horses have explored the association between dominance rank and body condition explicitly.

It is becoming increasingly important to understand the factors influencing body condition in domestic horses (*Equus caballus*) due to the increasing prevalence of obesity (Thatcher et al., 2008; Wyse et al., 2008; Thatcher et al., 2012; Giles et al., 2014) and obesity-associated disorders such as laminitis (Menzies-Gow et al., 2010) in the domestic equine population. Where horses are kept outdoors in groups, their social status could potentially determine access to forage resources (Rands et al., 2006; Ingólfsdóttir and Sigurjónsdóttir, 2008), especially during winter months (Appleby, 1980). A greater understanding of how individual behavioural and social factors influence body condition may be important for understanding determinants of health, not solely in horses but also in other managed species.

Whilst previous studies have explored the influence of physical factors upon dominance rank in horses (Houpt et al., 1978; Rutberg and Greenberg, 1990), this has not been considered *vice versa*, nor the direction of association considered; presumably because many physical attributes such as height and body size do not change much once fully grown, and therefore cannot change in response to a change in rank. Body condition may be more strongly determined by individual behavioural differences and is in this respect very different from size or height. It is not a fixed physical characteristic and can vary on a relatively short-term basis (Dugdale et al., 2010).

Our dataset also allows an exploration of the general hypothesis proposed by Maynard-Smith and Brown (1986), which states that a certain amount of natural phenotypic variation might aid herd cohesion and stability through reducing costly conflict behaviour. They argue that the lowest levels of conflict and aggression should be among animals with larger amounts of phenotypic variation, due to dominance relationships being contested less often, presumably as more obvious visual asymmetries between individuals can act as a cue for competitive ability (Arnott and Elwood, 2009).

Our study of domestic, outdoor-living horses investigates the association between body condition and dominance rank. Age and height are considered as confounders given their potential association with both dominance and body condition (Šárová et al., 2013).

The aims of our study were: (1) to explore the association between dominance and body condition in outdoor group-living domestic horses; and (2) to assess whether herds that are more similar in body condition, height or age have higher levels of displacement interactions or interactivity.

## 2 Materials and methods

### 2.1 Animals and inclusion criteria

We recruited 203 outdoor-living domestic horses across 42 separate herds, from a sampling frame of local owners and charitable organisations in North Somerset, Gloucestershire, Warwickshire, Norfolk and Essex. The term horses will refer to both domestic horses and ponies (*E. caballus*) throughout the manuscript. Most of these animals were based at UK charities or in herds already known to us. All owners had consented to participate. We were able to establish a dominance rank for 194 individuals, and these made up the final sample size used for analysis. Inclusion criteria specified that all individuals had to have lived together for a minimum of one month prior to inclusion in the study and had to be living outdoors in a group for 24 h a day during the study. This was an effort to ensure that dominance relationships were relatively stable and established. No groups containing mature stallions were included. Sex was recorded, but there was no apparent different in dominance status between geldings and mares and so these results are not presented. The herd size was limited to between two to eleven individuals, based on the feasibility of recording displacement interactions with a video camera (for details see ‘descriptive statistics’). In order to fully explore the effect of age within the analysis, there was no age restriction on study animals. Study horses were also a mix of different breeds.

### 2.2 Study design

The study was conducted during March 2012, before spring grass began to grow. This was when the least natural forage was likely to be available and food resource competition highest. Some study herds were being provisioned with supplementary hay, but despite this any differences in body condition relating to dominance were most likely to be apparent at this time of year in outdoor-living animals. A cross-sectional study design was used, with a cluster sampling strategy, where herds were randomly selected from the sampling frame and all horses measured in that herd.

The explanatory variable of interest was dominance rank. The outcome variable was body condition score (and also subsequent obesity, defined below). Age and height were recorded as potential confounders or effect modifiers. Age was measured in years and months, based on owner information, and height was measured at the withers (highest point of the shoulder, in cm) using a measuring stick with spirit level.

The work was approved by the University of Bristol Ethical Review Group (University Investigation Number UB/10/049), and complies with ASAB/ABS Guidelines for Use of Animals in Research.

### 2.3 Assessing food-related dominance rank

To assign a dominance rank, a simple feeding trial test was performed within the usual field enclosure of the study subjects. Each individual in a herd had an individual portion of food allocated to it, so there was the same number of portions as animals. If the herd did not already have an ad libitum hay supply, this portion was a small amount of hay, but if there was already hay present, this portion was a bucket containing a handful of low energy chaff-based feed (around 100 g).

To conduct the feeding trial test, all the individual portions were placed in the normal living enclosure of the animals at the same time (distributed quickly), at least one horse length apart (to minimise the risk of injuries from kicking). The horses were present whilst the food piles or buckets were distributed, but this was done quickly in an area of the paddock which was easy to film and not too wet (to minimise slipping). Horses were quick to approach, if not already present in the location where food was distributed. Every horse had an individual bucket or pile to feed from initially, but was able to move freely between food portions. When the horses began to finish their own pile of food, they moved to investigate the piles or buckets still being eaten by others. These movements and displacement interactions were filmed from the moment the first horse approached a pile of feed or a bucket, until all of the food was finished. The total length of feed trials varied between 1.03 min and 8.30 min, with a mean of 3.38 min.

Displacement interactions were recorded and coded from the videos using Noldus *Observer* XT 10 (Noldus Information Technology, Wageningen, Netherlands). A ‘displacement’ was defined as one horse moving towards another and the second individual moving away. Often the horse would have its ears back against the head with its neck outstretched. Occasionally the first horse reversed into the second using a threat to kick. Regardless of the approach, a displacement occurred when the second horse moved away. The number of displacements received by each individual was recorded for each herd dyad. Displacements were recorded for both the animal being displaced and the animal doing the displacing. All videos were coded by the principal investigator (SG). Videos were watched the same number of times as there were number of horses in the herd, so that each animal was individually watched to minimise coding errors.

Dominance rank was calculated according to the system described by Appleby (1980). If the number of displacements towards another herd individual was greater than the number of displacements received by that same individual then the individual was considered to be dominant. Individuals were then ranked according to the number of horses they were dominant over and dominated by. If two horses were identical, then they were given the same dominance rank, with no assumption of linearity.

To allow a comparison between herds of different sizes this final Appleby (Appleby, 1980) rank score (*a*) was adjusted to correct for herd size (*h*), using:

$$\text{adjusted}\;\text{dominance}\;\text{rank}=1-\frac{a-1}{h-1}$$

This ‘adjusted dominance rank’ was the outcome variable used in the statistical analysis, referred to as dominance rank. Note that nine individuals did not interact at all with others during the feed trial test and so these were not given a dominance rank as this could not be determined. The final sample size where dominance was considered was therefore 194.

Measures of interactivity and total number of displacements were also recorded during the video analysis, for an exploration of Maynard-Smith and Brown's (1986) hypothesis. Interactivity rank was the rank of the individual within the herd, when ordered according to the total number of interactions that individual was involved in during the feed trial (either aggressive or submissive), where the most interactive member of the herd was given rank 1. This was then adjusted for herd size in the same way as dominance rank above. Displacement was defined as the number of wins (displacements of another individual) per minute during the feed trial. By using a per-minute measure this controlled for the total length of feed trial which was variable between herds.

### 2.4 Assessing body condition

The outcome variable, body condition score (BCS), was measured using the Kohnke (Kohnke, 1992) interpretation of the Henneke et al. (1983) 9 point scale. Measurements were taken in the field just before the dominance trial was conducted. Six areas of the horse were given a score between 0 and 9 (neck, shoulder, withers, ribs, back, rump) and the mean score taken by dividing by six. This was done mostly visually but also by touch; the horses were all familiar with being handled. The final score was then rounded up to the nearest half measure. Each study horse or pony was scored by a single trained observer (SLG).

Obesity status was defined as a binary outcome variable, obese/non-obese, where obese was classified as a BCS of 7 or above (Dugdale et al., 2011).

### 2.5 Statistical analysis

All data analysis was conducted using *Stata* 12.1 (Statacorp, College Station, Texas, USA). Dominance rank was first assessed for a univariable association with body condition score using mixed effects linear regression, where the clustered study design was controlled for by including herd group as a random effect. Dominance rank was also tested for an association with obesity (obese/non-obese) using mixed effects logistic regression, again including herd group as a random effect. Note that dominance rank was normally distributed.

The potential confounding factors of age and height were assessed for a univariable association with dominance rank and also the outcome variables body condition score and obesity; again using linear regression and logistic regression. Non-linear quadratic relationships with age and height were considered by comparing models both with and without a quadratic function using a likelihood ratio (LR) test.

To explore whether herds that were more similar in body condition, height or age had a greater amount of within herd interactivity, the within-herd standard deviations in body condition, height and age were calculated as a measure of within-herd variance. These standard deviation measures of within-herd variance were then plotted against mean herd displacement (wins per minute) and total herd interactivity, defined as the total number of interactions within a herd (wins or losses). These relationships were then examined for a statistical association using linear regression, controlling for herd size.

If height or age was associated with both the explanatory (dominance rank) and the outcome variable (body condition score), it was considered as a potential confounder. Multivariable analysis using a mixed effects linear regression model was used to investigate confounding effects and calculate non-confounded estimates. Biologically plausible interactions, age and height, age and body condition, and height and body condition were also considered at this multivariable analysis stage.

## 3 Results

### 3.1 Descriptive statistics

The mean body condition score of the outdoor living study population was 5.53 ± 0.07 (unless otherwise stated, SEM given). The prevalence of obesity (BCS ≥ 7/9) was 17.24%. The mean age was 11.6 ± 0.5 years, ranging from 6 months to 35 years. The mean height was 132 ± 2 cm, ranging from 71 cm to 183 cm. The mean herd size was 5.8 ± 0.2, ranging from 2 to 11. 47% of study animals were mares and 53% were geldings.

The variation in body condition, age and height was greater between than within herds (see Supplementary information Table 1), suggesting that individuals that are kept together are likely to be more similar than average with relation to these characteristics. There was no relationship between the mean time spent feeding at the buckets and the number of interactions an individual engaged in (*F* = 0.87, *p* = 0.60, Supplementary Fig. 1), indicating that the absolute length of feed trial is unlikely to have influenced the displacement results. There was also no evidence of an association between the mean time spent eating in each herd and the number of interactions in that herd (*t* = 0.18, *p* = 0.88, Supplementary Table 2).

### 3.2 Associations with body condition score

Associations with body condition score, controlling for herd group are summarised in Table 1. There was strong evidence of a univariable association between adjusted dominance rank and body condition score (*Z* = 3.42, *p* = 0.001), as dominance rank increased, body condition score increased.

Height showed evidence of association with body condition score (Table 1, *Z* = −2.33, *p* = 0.02) where smaller individuals had a higher body condition score. Age showed no evidence of association with body condition score (Table 1, *Z* = 0.86, *p* = 0.39).

### 3.3 Associations with obesity

Table 2 shows associations with obesity (obese/non obese) controlling for herd group. Adjusted dominance rank was strongly associated with obesity (*Z* = 2.72, *p* = 0.007), where dominance rank was 11.82 (95% CI 1.99–70.05) times higher in obese compared to non-obese animals.

Height showed strong evidence of association with obesity (*Z* = −2.82, *p* = 0.005), where smaller individuals were more likely to be obese. Age showed no evidence of association with obesity (*Z* = −0.16, *p* = 0.87).

### 3.4 The relationship between age and height and dominance rank

**Fig. 1:**
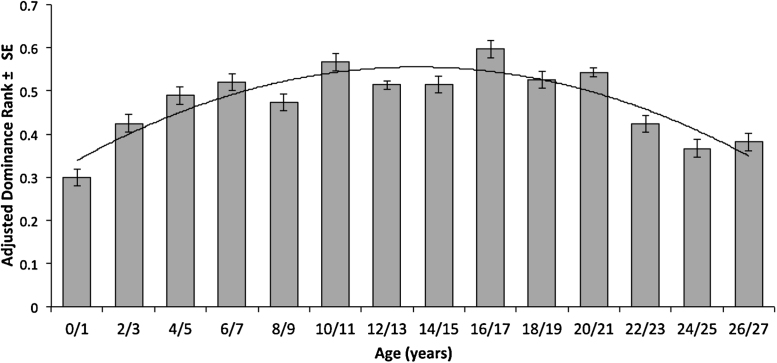
The quadratic relationship between age and dominance rank (adjusted for herd size, *d*). The line represents the quadratic equation age = −0.005*d*^2^ + 0.0757*d* + 0.2688. *R^2^* = 0.79094.

Age had a strong quadratic association with adjusted dominance rank (Likelihood Ratio Test $\chi_{193}^{2}=13.64$, *p* < 0.001) where middle-aged individuals were likely to have a higher dominance rank than very young or very old individuals (Fig. 1). There was no evidence of an association between height and dominance rank (*Z *= 0.38, *p* = 0.70).

#### 3.4.1 Similarity between individuals within herds

**Fig. 2:**
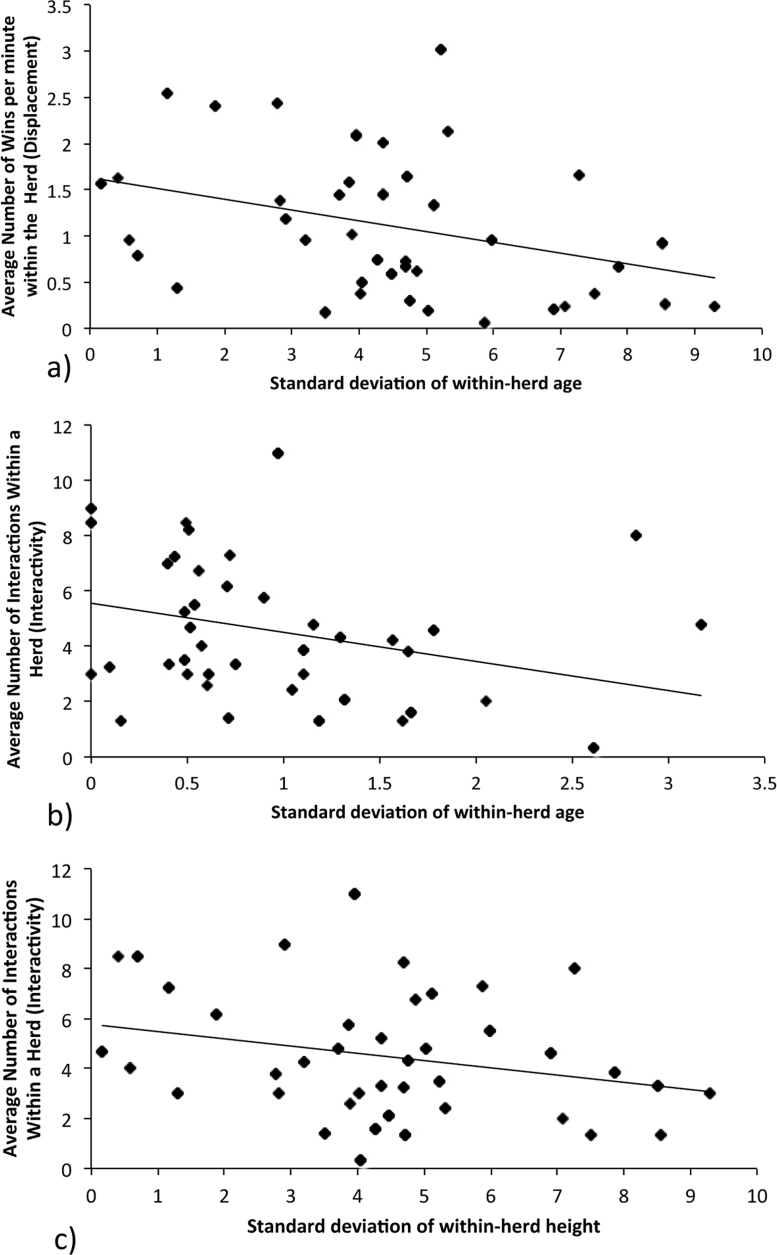
The relationship between within-herd variation in age and height and average number of wins per minute and total herd interactivity. (a) Herd displacement (number of wins) and standard deviation of within-herd age; (b) herd total interactivity and standard deviation of within-herd age; (c) herd total interactivity and standard deviation of within-herd height.

Within-herd variation (standard deviation) in body condition was not associated with total herd interactivity (*t*_202_ = −1.01, *p* = 0.32) or number of displacements (*t*_202_ = −0.89, *p* = 0.37). However there was evidence of a negative association between the within-herd variation in age and mean number of displacements within the herd (Fig. 2a, *t*_202_ = −2.49, *p* = 0.01), as the within-herd standard deviation in age increased, within-herd displacement decreased. Variation in age was also associated with lower within-herd interactivity (Fig. 2b, *t*_202_ = 3.36, *p* = 0.001).

Variation in height was not associated with within-herd displacement (*t*_202_ = −0.39, *p* = 0.70) but did show evidence of association with interactivity (Fig. 2c, *t*_202_ = −4.25, *p *< 0.001), where herds more similar in height were more interactive.

### 3.5 Similarity in age and height and the effect on body condition score

The within-herd standard deviation of age tended to be associated with individual body condition score (*t*_202_ = 1.78, *p* = 0.07) and within-herd standard deviation of height showed no evidence of association with individual body condition score (*t*_202_ = −0.30, *p = *0.76).

### 3.6 Multivariable analysis

As described, age showed a quadratic relationship with dominance rank but there was no evidence of an association between age and body condition score (Table 1). Height showed no evidence with dominance rank, but strong evidence of association with body condition score. This meant that it was not necessary to treat either of these variables as confounding measures. No significant interactions between height, age and body condition were found (Table 3), suggesting that the relationship between dominance rank and body condition score is entirely independent of age and height.

## 4 Discussion

Our study presents strong evidence that dominance status may be associated with body condition in domestic outdoor-living horses, and this association appears to be entirely independent of both age and height, both of which have been shown to be important determinants of dominance in horses in previous studies (Rutberg and Greenberg, 1990; Sigurjónsdóttir et al., 2003).

The feed trial method used here allowed for a competitive, food-related assessment of dominance and related behaviours. It is not known whether the dominance seen in this feed trial technique would mirror that seen in an extended foraging scenario using field observations. This feed trial would certainly be worth validating against a field observation measure. A strong correlation between field-observed dominance and a dyad-based feed trial has previously been shown in a small study on groups of young geldings (Ahrendt and Christensen, 2012). Another obvious limitation of this observational technique is the varying length of each feed trial, which was dependent on how long it took for the food in the buckets to be eaten. This didn’t have any obvious effect on the number of interactions seen in this study, potentially because horses did not begin to move and interact until a relatively dominant individual had finished feeding from its initial bucket. The largest number of interactions therefore occurred at the end of the trial, regardless of the total trial length.

Previous authors have speculated that the mechanisms behind observed differences in size in relation to dominance rank (where more dominant animals are larger) might be due to differences in the foraging behaviour of dominant versus more subordinate individuals (McGreevy and Burgess, 2005; Ingólfsdóttir and Sigurjónsdóttir, 2008). It has been suggested that a higher dominance rank results in a greater energetic intake during foraging, particularly during winter months when resources are scarce (Appleby, 1980; McGreevy and Burgess, 2005; Ingólfsdóttir and Sigurjónsdóttir, 2008). However other studies have found no obvious association between dominance and feeding behaviour itself (Feist and McCullough, 1976). This study confirms that these two factors are indeed associated empirically, although additional investigations are required to further elucidate the mechanisms driving this relationship and the direction of association. It may be that animals with a greater initial body condition may simply have a competitive advantage (Rands et al., 2006).

Alongside body condition, our results show a strong association between dominance rank and obesity (BCS ≥ 7/9), where dominant individuals appear to be at a higher risk of obesity. This is the first study to demonstrate the potential influence of behavioural factors upon clinical obesity risk in socially living domestic horses, though as noted, the direction of causal association remains unknown. Regardless of the direction of association, results here suggest that special attention should be directed towards correctly managing the intake of more dominant animals within a domestic herd, to prevent obesity in these higher risk animals.

The clinical importance of understanding factors influencing obesity in domestic horses has only recently been recognised and risk factors influencing obesity susceptibility in horses are still not well understood. Recent studies suggest that supplementary diet and exercise levels are not solely responsible for body condition variation, especially in outdoor living animals (Giles et al., 2014). Many previous studies investigating dominance in horses have been small in sample size, and have tended to explore a large behavioural repertoire in feral, or related harem of animals in captivity (Clutton-Brock et al., 1976; Berger, 1977; Rutberg and Greenberg, 1990; Sigurjonsdottir et al., 2003). The statistical power to detect associations between dominance and explanatory variables may therefore be low, potentially explaining contrasting results over the decades regarding the variables associated with dominance in horses.

Several previous authors have suggested that size, weight or height may be an important determinant of dominance (Clutton-Brock et al., 1976; Houpt et al., 1978; Ingólfsdóttir and Sigurjónsdóttir, 2008), presumably because a larger size affords a greater competitive advantage in a resource conflict situation (Maynard-Smith and Brown, 1986). Height was not associated with dominance rank in this study, however height was associated with body condition, where smaller individuals generally had a higher body condition score. This perhaps represents the known breed predisposition to obesity of native UK pony types (Giles et al., 2014; Robin et al., 2014). Vervaecke et al. (2005) found that fatness, as opposed to overall size or height, was the strongest predictor of dominance rank in bison and our results here suggest this is also the case in outdoor-living domestic horses.

Age has been recognised as an important predictor of dominance in equines (Rutberg and Greenberg, 1990; Van Dierendonck et al., 1995) and our results support this. Age showed a strong quadratic association with dominance, where middle-aged individuals were likely to have the highest dominance rank. In many previous studies in which age is significantly associated with dominance, authors report a linear association (Keiper, 1988; Van Dierendonck et al., 1995; Heitor et al., 2006). Potentially previous studies have lacked either the statistical power or a sufficient age range within the study population to detect a quadratic relationship, or simply a quadratic relationship was not considered or present.

A quadratic relationship with age is common for many physiological parameters, which peak during the active reproductive life of the animal. As body condition is likely to be closely related to reproductive fitness in horses and horses do not reach full size or sexual maturity until around five years of age (Goodwin, 2007), a lower dominance rank in immature animals might be expected. Alongside this, these results suggest that older equines over 20 years of age are also likely to have a lower dominance rank, again potentially associated with a lower physiological and reproductive fitness.

Despite the quadratic association between age and dominance there was no similar quadratic association between age and body condition score, i.e. middle-aged individuals did not obviously have a higher body condition score. Potentially, herds more similar in age or body condition score are likely to be kept together which masked any such association. This may well be the case in our study population, where the within-herd variation in age was much lower than the between herd variation in age and the same was true for body condition score, (see supplementary table).

Herds which had less phenotypic variation within age and height had higher levels of interactivity among herd individuals within the feeding trial, this fits our hypothesis and with the predictions made by Maynard-Smith and Brown (1986). Displacement was also higher in herds where individuals were more similar in age. Notably, within herd variation in body condition did not show this same relationship, individuals more similar in body condition were not necessarily more interactive. Potentially this is because our study used well established social groups or body condition may be a more obvious visual cue for competitive ability in horses (Arnott and Elwood, 2009), therefore where body condition variation within a herd exists, this may act to reduce the levels of interaction and displacement observed.

These results could have important management implications for domestic horses, which are often kept in same age or same breed groups, particularly on stud or racing yards. Very young animals are often kept in larger outdoor groups, and our study results indicate that younger equines are more interactive (*p* = 0.001) than older equines. Managing horses in mixed groups (in terms of age, size and breed) may result in fewer interactions and thus less concern over injuries for horse owners. In line with this, results of a previous study indicated that mares that were more different in age were more likely to associate and engage in friendly interactions (Heitor et al., 2006).

## Conflict of interest

The authors declare no competing interests. Patricia A Harris is an employee of WALTHAM Centre for Pet Nutrition.

## Figures and Tables

**Table 1:** Associations between explanatory variables and the outcome body condition score using mixed effects linear regression. Herd group was included in each model as a random effect.

Explanatory variable	*β*	SE	95% CI	*Z*	*p*	*n*
Social indices						
Dominance rank	0.92	0.27	0.39–1.44	3.42	0.001	194

Other variables						
Age	0.01	0.01	−0.01 to 0.03	0.86	0.39	202
Height	−0.07	0.03	−0.13 to −0.01	−2.33	0.02	203

**Table 2:** Associations between explanatory variables and the outcome obesity (obese/non-obese) using mixed effects logistic regression. Herd group was included in each model as a random effect.

Explanatory variable	Odds ratio	SE	95% CI	*Z*	*p*	*n*
Dominance rank	11.82	10.73	1.99–70.05	2.72	0.007	194

Other variables						
Age	1.00	0.03	0.95–1.05	−0.16	0.87	202
Height	0.81	0.06	0.70–0.94	−2.82	0.005	203

**Table 3:** The association between the potential interaction terms, age and height, upon body condition score and dominance rank, tested using multivariable linear regression models. The three models contain different combinations of the explanatory variables body condition score, age and height, and dominance rank is always the outcome variable.

Explanatory model terms	*β*	SE	95% CI	*Z*	*p*
BCS and height					
BCS	0.15	0.10	−0.03 to 0.34	1.60	0.11
Height	0.05	0.04	−0.04 to 0.13	1.12	0.26
BCS × height	−0.007	0.008	−0.02 to 0.008	−0.94	0.35
Constant	−0.48	0.55	−1.55 to 0.59	−0.88	0.38

BCS and age					
BCS	0.06	0.03	−0.003 to 0.13	1.85	0.07
Age	−0.0001	0.01	−0.03 to 0.03	−0.01	0.99
BCS × age	0.0001	0.002	−0.004 to 0.004	0.05	0.96
Constant	0.14	0.18	−0.22 to 0.50	0.77	0.44

Height and age					
Height	−0.01	0.01	−0.04 to 0.01	−1.18	0.24
Age	−0.02	0.01	−0.04 to 0.003	−1.65	0.10
Height × age	0.001	0.0009	−0.0002 to 0.003	1.76	0.08
Constant	0.66	0.17	0.34–0.99	3.97	<0.001
