# Erratum to “Dominance rank is associated with body condition in outdoor-living domestic horses (*Equus caballus*)” [Appl. Anim. Behav. Sci. 166 (2015) 71–79]

**DOI:** 10.1016/j.applanim.2015.08.028

**Published:** 2015-10

**Authors:** Sarah L. Giles, Christine J. Nicol, Patricia A. Harris, Sean A. Rands

**Affiliations:** a University of Bristol, School of Veterinary Science, Langford, Bristol BS40 5DU, UK; b University of Bristol, School of Biological Sciences, Tyndall Avenue, Bristol BS8 1TQ, UK; c WALTHAM Centre for Pet Nutrition, Equine Studies Group, Freeby Lane, Waltham-on-the-Wolds, Melton Mowbray, Leicestershire LE14 4RT, UK

This is to inform the readers that the acknowledgement to BBSRC ‘Open Access funded by Biotechnology and Biological Sciences Research Council’ was captured in the online version and not in print.

The publisher would like to apologise for any inconvenience caused.

