# Supplementary material for: Dominance rank is associated with body condition in outdoor-living domestic horses (Equus caballus)
Source: Appl Anim Behav Sci. 2015 May;166:71–9. doi: 10.1016/j.applanim.2015.02.019 (PMC4407901; doi:10.1016/j.applanim.2015.02.019)
Supplement: Supplementary file 1 [file mmc1.docx]

## Supplementary Information

**Supplementary Table 1: Analysis of Variance showing between and within group variation of body condition, age and height**

| ***Variable*** | ***Between Herd Variation (MS)*** | ***Within Herd Variation (MS)*** | ***df. (between, within)*** | ***F*** | ***p*** |
| --- | --- | --- | --- | --- | --- |
| ***BCS*** | 1.41 | 0.84 | 40, 162 | 1.68 | 0.01 |
| ***Age*** | 194.65 | 25.70 | 40, 161 | 7.57 | <0.001 |
| ***Height*** | 1010.55 | 239.36 | 40, 162 | 17.10 | <0.001 |

**Supplementary Figure 1: Graph showing the mean total time spent feeding from the buckets during the feed trial against total number of interactions for each individual (F=0.87, *p*=0.60)**

**Supplementary Table 2: Table showing herd size, mean time spent feeding from buckets and the mean number of interactions by herd. There is no relationship between mean time spent feeding from bucket (giving an indication of length of feed trial) and mean number of interactions.**

| ***Herd Number*** | ***Herd Size*** | ***Mean Time Spent Eating from Bickets*** | ***Mean Number of Interactions*** |
| --- | --- | --- | --- |
| 1 | 8 | 190.26 | 3.88 |
| 2 | 11 | 130.55 | 2.09 |
| 3 | 5 | 209.50 | 9.00 |
| 4 | 3 | 121.72 | 4.00 |
| 5 | 4 | 157.38 | 3.25 |
| 6 | 5 | 91.77 | 6.20 |
| 7 | 4 | 80.70 | 5.25 |
| 8 | 6 | 109.92 | 3.33 |
| 9 | 3 | 356.11 | 3.00 |
| 10 | 3 | 168.04 | 1.33 |
| 11 | 6 | 75.17 | 3.03 |
| 12 | 4 | 52.89 | 3.50 |
| 13 | 5 | 61.68 | 3.80 |
| 14 | 4 | 141.08 | 5.75 |
| 15 | 4 | 61.55 | 4.25 |
| 16 | 3 | 96.68 | 3.33 |
| 17 | 2 | 81.52 | 3.00 |
| 18 | 3 | 224.31 | 4.67 |
| 19 | 5 | 109.63 | 2.60 |
| 20 | 8 | 105.66 | 6.75 |
| 21 | 2 | 176.02 | 8.50 |
| 22 | 3 | 120.49 | 1.33 |
| 23 | 6 | 172.89 | 8.50 |
| 25 | 3 | 60.49 | 1.33 |
| 26 | 4 | 130.21 | 3.00 |
| 27 | 7 | 328.50 | 2.43 |
| 28 | 10 | 208.77 | 4.60 |
| 29 | 5 | 242.81 | 1.60 |
| 30 | 5 | 104.10 | 4.80 |
| 31 | 5 | 207.26 | 1.40 |
| 32 | 5 | 89.34 | 4.80 |
| 33 | 4 | 138.21 | 7.25 |
| 34 | 5 | 92.76 | 8.00 |
| 35 | 6 | 209.13 | 5.50 |
| 36 | 5 | 212.26 | 3.00 |
| 37 | 10 | 138.40 | 11.00 |
| 38 | 4 | 330.31 | 8.25 |
| 39 | 7 | 388.66 | 7.29 |
| 40 | 3 | 301.35 | 0.33 |
| 41 | 2 | 219.60 | 2.00 |
| 42 | 6 | 414.59 | 4.33 |
